# Actual fusion efficiency in the lipid mixing assay - Comparison between nanodiscs and liposomes

**DOI:** 10.1038/srep43860

**Published:** 2017-03-07

**Authors:** Claire François-Martin, Frédéric Pincet

**Affiliations:** 1 Laboratoire de Physique Statistique, Ecole Normale Supérieure, Paris Sciences et Lettres Research University, 75005, Paris, France; 2 Laboratoire de Physique Statistique, Université Paris Diderot Sorbonne Paris Cité, 75005, Paris, France; 3 Laboratoire de Physique Statistique, Sorbonne Universités, Université Pierre et Marie Curie, Univ Paris 06, CNRS, 75005, Paris, France

## Abstract

Lipid exchange occurs between membranes during fusion or active lipid transfer. These processes are necessary *in vivo* for the homeostasis of the cell at the level of the membranes, the organelles and the cell itself. They are also used by the cell to interact with the surrounding medium. Several assays have been developed to characterize *in vitro* these processes on model systems. The most common one, relying on fluorescence dequenching, measures lipid mixing between small membranes such as liposomes or nanodiscs in bulk. Usually, relative comparisons of the rate of lipid exchange are made between measurements performed in parallel. Here, we establish a quantitative standardization of this assay to avoid any bias resulting from the temperatures, the chosen fluorescent lipid fractions and from the various detergents used to normalize the measurements. We used this standardization to quantitatively compare the efficiency of SNARE-induced fusion in liposome-liposome and liposome-nanodisc configurations having similar collision frequency. We found that the initial yield of fusion is comparable in both cases, 1 per 2–3 million collisions in spite of a much larger dequenching signal with nanodiscs. Also, the long-term actual fusion rate is slightly lower with nanodiscs than in the liposome-liposome assay.

*In vivo*, lipid mixing and exchange constantly occur between cells and/or organelles. Fusion is a common process that merges two membranes thereby mixing their initially separated lipids^1^,^2^. Direct lipid transport is now frequently described as a way to specifically exchange lipids between membranes^3^,^4^. The pioneering work of Pagano *et al*. in 1981 presented a simple approach to assess the amount of lipid transfer between liposomes *in vitro*^5^. They initially studied the calcium induced fusion of negatively charged membranes. The principle of this technique, often referred to as the lipid mixing assay, is to mix two sets of membranes: a non-fluorescent one and another one, fluorescent, that contains quenched fluorescent lipids. In the initial work, the membranes were liposomes and the fluorescent groups were Nitro-2,1,3-benzoxadiazole-4-yl (NBD) and Lissamine Rhodamine B (Rho). Förster Resonance Energy Transfer^6^ (FRET) can occur between these dyes at sufficiently high concentration on the membrane. Then, when NBD, the donor, is excited, instead of the emission of a photon, energy is transferred to Rho, the acceptor, that emits a photon. Upon fusion between a fluorescent and a non-fluorescent membrane, the dyes are diluted according to the ratio between the areas of the two membranes. Upon dilution of the dyes, FRET is reduced. Hence, NBD fluorescence, initially low, increases as the dyes are dequenched during the many fusion events that occur in bulk. The extent of fusion is currently estimated by the addition, at the end of the experiment, of a detergent that disrupts the liposomes and provides a final fluorescence that is considered as the one after infinite dilution of the dyes.

This lipid mixing assay has been used in hundreds of publications, mainly to monitor fusion^5^,^7^,^8^. Rho and NBD still are the most common FRET pair used and usually, the donor and the acceptor each represent 1.5% of the total lipid in the fluorescent membranes. The fusion of membranes other than small liposomes, *e.g*. Nanodiscs^9^,^10^ or Giant Unilamellar Vesicles^11^, has also been investigated with the lipid mixing assay. When the areas of the two membranes is very different, e.g. liposome-nanodisc, the donor membrane must be the smallest one to ensure maximum dilution and maximum fluorescence increase.

The lipid mixing assay monitors the transfer of lipids from the donor to the acceptor membrane but, a priori, does not distinguish between fusion, hemifusion or mere lipid transport without membrane sharing. Distinguishing between these various processes requires additional experiments/techniques such as content mixing, or cryo-EM observation of the samples.

Because, no matter the mechanism, lipid transfer is a rare event, the fluorescence increase has to be monitored over tens of minutes, typically one hour, to obtain a significant signal. Even on these time sales, in the vast majority of the reports that used the lipid mixing assay, only a fraction of the donor membranes underwent fusion. In the rare cases in which sequential fusion events occurred, each successive fusion started with a lower concentration and led to a lesser dilution than the previous one. Hence, the fluorescence increase is reduced as membrane mixing extends between the liposomes. With this in mind, the notion of average “rounds of fusion” was introduced and brought a better view of the average number of successive fusion events that the donor membranes underwent^12^.

Because the analysis of the fluorescence increase curves strongly depends on many factors such as the normalization by the detergent or the temperature, usually the lipid mixing assay is used to compare fusion rates between experiments performed under exactly the same conditions. Here, our aim is to bring a more standardized analysis of the mixing assay by providing an absolute lipid mixing extent and allowing a more direct comparison between various conditions. We use this standardization to more accurately evaluate the fraction of fused donor membranes in each situation and apply it to compare liposome-liposome and liposome-nanodisc fusion.

## Results

### Final lipid fluorescence depends on the membrane disrupting detergent

We used liposomes primarily made of POPC. As in the vast majority of reported experiments with the lipid mixing assay, fluorescent membranes always contained the same small fraction of donor, NBD-DOPE, and acceptor, Rho-DOPE, fluorescent lipids. We monitored the fluorescence of NBD-DOPE in a 96-well plate reader. To be able to go to low fractions of fluorescent lipids and still obtain a fluorescence signal, we worked at 18 mM lipids. Liposomes were prepared by extrusion (see Experimental Section), they were monodisperse and their diameter, obtained by Dynamic Light Scattering, was ~80 ± 20 nm. Using these liposomes, we evaluated (i) the relevance of using detergent as a reference for infinite dilution, (ii) the effect of temperature on the infinite dilution fluorescence value and (iii) an absolute quantification of the level of fusion with the ratio between the donor and acceptor membranes areas.

**Figure 1:**
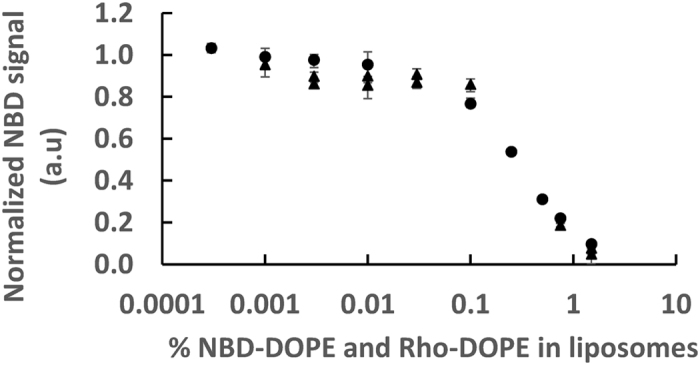
Infinite dilution of the fluorescent lipids. Intrinsic fluorescence of NBD-DOPE, i.e. total fluorescence divided by the quantity of dyes, was measured for various fractions of fluorescent lipids (NBD-DOPE and Rho-DOPE being both equal). The circles are data points with only fluorescent liposomes at 18 mM lipids. Triangles were obtained from various experiments in which there was a mixture of fluorescent and non-fluorescent liposomes at ratios varying from 1:2 to 1:10; the total lipid concentration also being 18 mM. As fluorescent lipid fractions decrease, FRET between NBD and Rhodamine is reduced. The plateau, corresponding to the absence of FRET, i.e. infinite dilution, is reached for fluorescent lipids fraction bellow or equal to 0.01%, i.e. for ~1 fluorescent lipid per liposome or less. 1 was set as the average of the data points below 0.01% from the purely fluorescent lipids. Error bars are the minimum and maximum values from 4 different wells (often smaller than the marker). The temperature is set at 37 °C.

**Figure 2:**
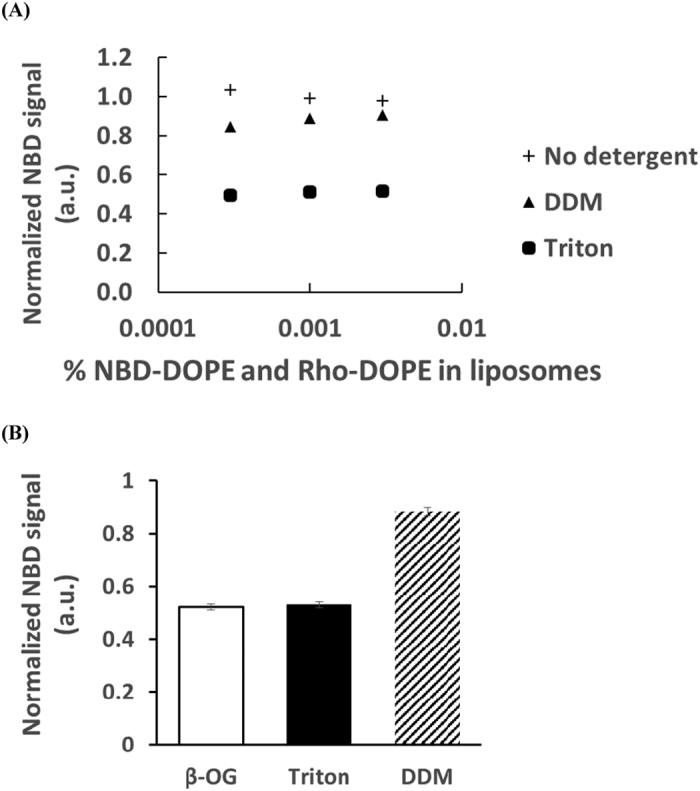
Normalization with various detergents. (**A**) Fluorescence after addition of the same volume of buffer or detergent solution (final concentration is 0.83% wt/wt for DDM and 0.67% vol/vol for Triton) to a liposome solution with 18 mM lipids. To ensure a complete dissociation of the liposomes and the absence of FRET within micelles, detergent was added up to 55 mM for Triton (more than 200 times its CMC) and up to 80 mM for DDM (more than 500 times its CMC), without any change in the outcome. Only fluorescent lipids concentrations below 0.01% have been tested because they provide fluorescence intensities close to that of infinite dilution (see Fig. 1). Fluorescence is decreased upon addition of detergent. The values are normalized by the average fluorescence after addition of buffer (crosses). All standard deviations are within the size of the markers. (**B**) Relative fluorescence intensities after addition of DDM, Triton or β-OG (final concentration 0.83% wt/wt, 0.67% vol/vol and 0.83% wt/wt respectively) to a liposome solution with 1 mM lipids and containing 1.5% NBD-DOPE and 1.5% Rho-DOPE. The values are normalized by the average fluorescence produced by DDM in panel A. Error bars are standard deviations on more than 50 wells. In both panels, the temperature is set at 37 °C.

In the lipid mixing assay, fluorescence after complete dequenching of the fluorescent lipids is estimated by adding detergent that disrupts the liposomes. The idea is to incorporate the lipids into detergent micelles thereby isolating the fluorescent dyes from one another. The resulting fluorescence is commonly considered as the reference for infinite dilution. We first tested the accuracy of this reference by actually diluting the fluorescent lipids in liposomes and evaluating their intrinsic fluorescence at infinite dilution without detergent. For that purpose, we changed the fraction of fluorescent lipids in the liposomes from 1.5% to 0.0003% for both NBD-DOPE and Rho-DOPE at 37 °C. The results are displayed in Fig. 1 (black circles). They show that complete dequenching is achieved below 0.01% of both fluorescent lipids, and that NBD-DOPE presents about 10% of its maximum fluorescence when NBD-DOPE and Rho-DOPE are incorporated at the commonly used fraction of 1.5%. In the course of other experiments, we have also tested solutions with mixtures of fluorescent and non-fluorescent liposomes. No significant difference was observed in this case (other data points in Fig. 1). We then investigated the ability of detergents to provide final fluorescence values close to that of infinite dilution in a liposome membrane. We tested n-dodecyl-β-D-maltoside (DDM) and Triton X-100 (Triton) at fluorescent lipid fractions for which infinite dilution is already reached in absence of detergent: 0.003%, 0.001% and 0.0003% of Rho-DOPE and NBD-DOPE (see Fig. 1). The results, displayed in Fig. 2A, show that 0.83% wt/wt DDM, i.e. 16 mM, (respectively 0.67% vol/vol Triton, i.e. 10 mM) provides close to 85% of the maximum signal (respectively 50%). The concentration of both detergents is well above their critical micelle concentration (CMC), at which they disrupt membranes, 0.15 mM (respectively 0.24 mM). In order for FRET to be entirely suppressed, lipids should, of course, be solubilized in micelles but, moreover, each fluorescent lipid should be incorporated in a different micelle. In theory, this requirement is met in all experiments presented here. The highest fluorescent lipid to detergent ratio is obtained with 1 mM lipid with 1.5% NBD-DOPE (15 μM) and 1.5% Rho-DOPE (15 μM). Since DDM micelles contain ~100 detergent molecules, there will be on average 7 lipid molecules and 0.2 fluorescent lipid molecule per micelle.

To confirm that there was less than one fluorescent lipid molecule per micelle, we added up to five times the initial amount of detergent, without any change in the outcome, meaning that the dyes were already diluted enough to avoid FRET (Fig. 2A). What is the origin of this difference between the detergents and why do they systematically lead to a lower fluorescence than the infinite dilution? We can only speculate but this difference is probably due to the local environment of the dyes that is different with the various detergents (larger micelles, more hydrophobic surrounding, etc…), and which could impact the quantum yield of NBD-DOPE.

### Fluorescence after detergent addition is lower than fluorescence at infinite dilution and depends on temperature

To check the validity of these values under usual experimental conditions, we also compared the fluorescence resulting from the disruption of liposomes with 1.5% of NBD-DOPE and Rho-DOPE (Fig. 2B). Because of the large absolute fluorescent dyes concentration (0.27 mM of NBD-DOPE and Rho-DOPE), it would require 9 M lipids to rearrange the fluorescent dyes in enough different liposomes to reach the threshold of infinite dilution (0.003% NBD-PE). Hence, it is not possible to measure the actual infinite dilution fluorescence under these conditions. However, the relative values of DDM and Triton remain the same suggesting the detergents have similar dequenching efficiencies as at lower NBD-DOPE and Rho-DOPE concentrations. The relative increase of fluorescence upon detergent addition can thus be considered as independent of the fluorescent lipid fraction at least up to 1.5%. In addition, β-OG, which is considered to costly to use in the lipid mixing assay, appears to have an efficiency close to that of Triton.

The primary consequence of this underestimate of the infinite dilution when adding detergent is an overestimate of the percentage of lipid mixing. A less effective detergent such as Triton or β-OG will artificially increase the percentage of variation due to lipid mixing, *e.g*. the same experiment will seem more efficient with Triton than with DDM.

**Figure 3:**
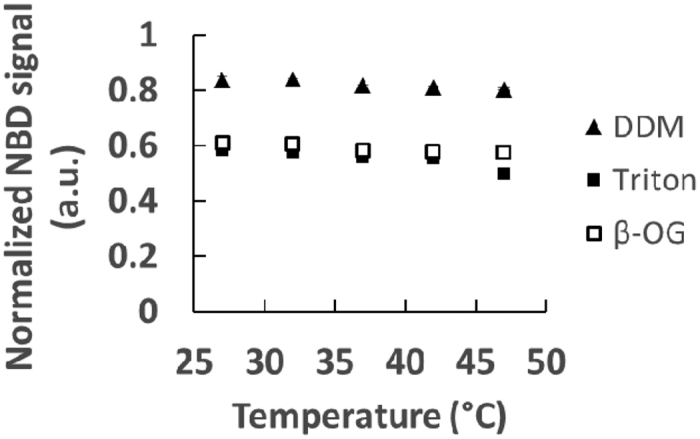
Detergent normalization with the temperature. Following the same procedure as that described in Fig. 2A for liposomes containing 0.001% NBD-DOPE and Rho-DOPE and a concentration of 18 mM lipids. This is done for a range of temperatures between 27 °C and 47 °C.

We also studied the fluorescence after detergent addition and compared it to that of infinite dilution at various temperatures by performing similar measurements between 27 °C and 47 °C. The results are displayed in Fig. 3. There is a small but visible effect of the temperature on the correction between the fluorescence after addition of detergent and the fluorescence at infinite dilution. The underestimate of the fluorescence at infinite dilution using that after detergent addition is more pronounced at higher temperatures. The ratios *α*_*det*_(*T*) of the real fluorescence at infinite dilution to that with detergent at temperature *T* are indicated in Table 1. Then, in any experiment, the fluorescence after infinite dilution in the liposome membrane, *F*_*∞*_, is directly obtained from the bulk fluorescence after detergent addition, *F*_*det*_, by:


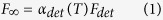


### Fraction of fused membranes deduced from fluorescence dequenching measurements

When analyzing lipid mixing experiments, the extent of the fluorescent lipids dilution, directly related to the extent of fusion, is commonly studied. To link these two parameters together, we have measured the normalized dequenching that would result from different extents of dilutions, *d*:


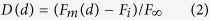


where *F*_*m*_(*d*) is the measured fluorescence for a dilution *d (i.e*. initial fraction/final fraction; d = 1 initially, and d = 2 when the dyes have been diluted twice) and *F*_*i*_ the initial fluorescence intensity at 1.5% fraction of fluorescent lipids, *i.e*. before lipid mixing (=*F*_*m*_(*1*)).

A standardization of the analysis of the lipid mixing assay by the determination of the real dequenching of the fluorescent lipid, *D*_*real*_, would make it easier to compare values between independent experiments. This standardization is now possible using the results presented above. During a kinetics experiment, the dequenching that is usually reported is normalized by *F*_*det*_, the fluorescence intensity after addition of detergent. It can be written: *D*_*m*_(*t*) = (*F*_*m*_(*t*) − *F(0*))/*F*_*det*_ where *F*_*m*_(*t*) is the fluorescence intensity measured at time *t*. Applying Eq. (1), the real dequenching normalized to infinite dilution is directly obtained by:


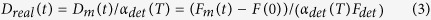


**Figure 4:**
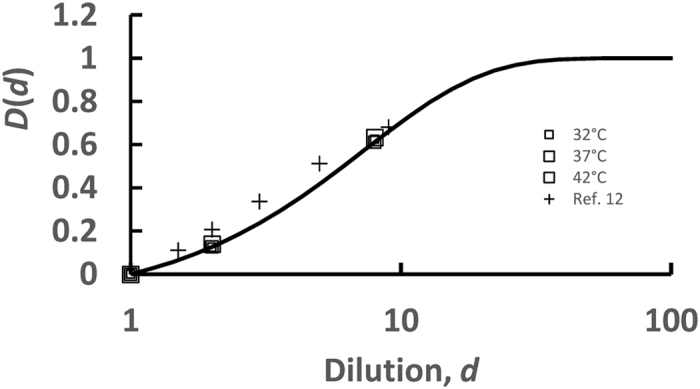
Increase of fluorescence upon dequenching after dilution of the dyes. Dequenching obtained from Eq. (2) using solutions containing liposomes with 1.5% (no dilution, *d* = 1), 0.75% (diluted twice, *d* = 2) and 0.19% (diluted 8 times, *d* = 8) NBD-DOPE and Rho-DOPE lipids. Dilution experiments were performed at 32 °C, 37 °C and 42 °C. The results do not depend on the temperature and are consistent with previous reports at 37 °C^12^ (crosses). The continuous line, represented between *d *= 1 and *d *= 100 corresponds to the fit obtained by using Eq. (3) between *d *= 1 and *d* = 8. Representing it until *d *= 100 was only done for the sake of clarity, to check that, indeed, this fit allowed to find that, from one point, further dilution does not lead to further dequenching, since the dyes are already sufficiently far from each other for FRET not to occur.

In order to estimate the fraction of fluorescent membranes that have been implicated in a fusion event, *R*, we have measured the dequenching *D(d*) corresponding to the standard initial fluorescent lipid concentration, 1.5%, by keeping the same total lipid concentration, and with NBD-DOPE and Rho-DOPE fractions equal to 1.5%, 0.75% (2-fold dilution) and 0.19% (8-fold dilution) in the temperature range 32 °C–42 °C (squares in Fig. 4). Experimentally, we observed that *D(d*) is independent of T and can be well approximated by:


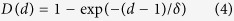


where *δ* is a characteristic decay dilution and is equal to 7.2 ± 0.2. Previous reports^12^ of dequenching after dilution are consistent with the ones we find here (other data points in Fig. 4).

If the average area of a non-fluorescent membrane is *λ* times that of the fluorescent membrane, the measured dequenching after all fluorescent membranes have fused is directly obtained from Eq.4 and given by:


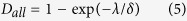


Most experiments are too short for a significant number of fluorescent membranes to undergo more than one round of fusion. However, in the rare cases in which several rounds of fusion occur, completing *n* rounds of fusion is equivalent to a dilution equal to *nλ* + 1 (*n* being an integer). Hence, the measured dequenching after *n* rounds of fusion is directly obtained from Eq. 5 and given by:


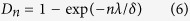


when the measured real dequenching, *D*_*real*_(*t*) (from Equation 3), is between *D*_*n*_ and *D*_*n+1*_, the average rounds of fusion per fluorescent membrane, *R*, can be well estimated by a linear dependence:


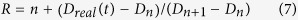


Clearly, this is a simplification because there will be a distribution of rounds of fusion between membranes. This distribution is difficult to predict because each fusion is different from the others mainly because of the initial distributions of the size of the involved membranes and of the number of proteins in each membranes. Hence *R* can only be considered as an indication of the extent of the fusion reaction.

### Liposome-liposome fusion vs. liposome-nanodisc fusion

This correction from Eq. 3 is presented on the example of two fusion experiments in Fig. 4A,B. In both experiments, soluble NSF attachment protein receptor (SNARE) induced membrane fusion. In the first one, two sets of fluorescent liposomes of similar sizes (~50 nm) were reconstituted (see Experimental Section) with either v-SNAREs (fluorescent v-liposomes; lipid to protein ratio: ~200) or t-SNAREs (non-fluorescent t-liposomes; lipid to protein ratio: ~400). The total lipid concentration was 3 mM and there were about 10 non fluorescent liposomes per fluorescent liposome. In the second one, a set of non-fluorescent t-liposomes containing t-SNAREs (similar to that in the first experiment) was mixed with 12 nm fluorescent v-nanodiscs containing v-SNAREs (lipid to protein ratio: 200). The total lipid concentration was 1.5 mM and there was about 1 t-liposome per v-nanodisc. Since both experiments were performed at 37 °C and initially normalized by the fluorescence after DDM addition, the real final dequenching is obtained with Eq. (3) through the reduction of the measured one by the same factor *α*_*det*_(*T*) = *α*_*DDM*_(37 °C). Hence, their relative rates remain the same: the rate of the liposome-liposome reaction seems much lower than that of the liposome-nanodisc one.

**Figure 5:**
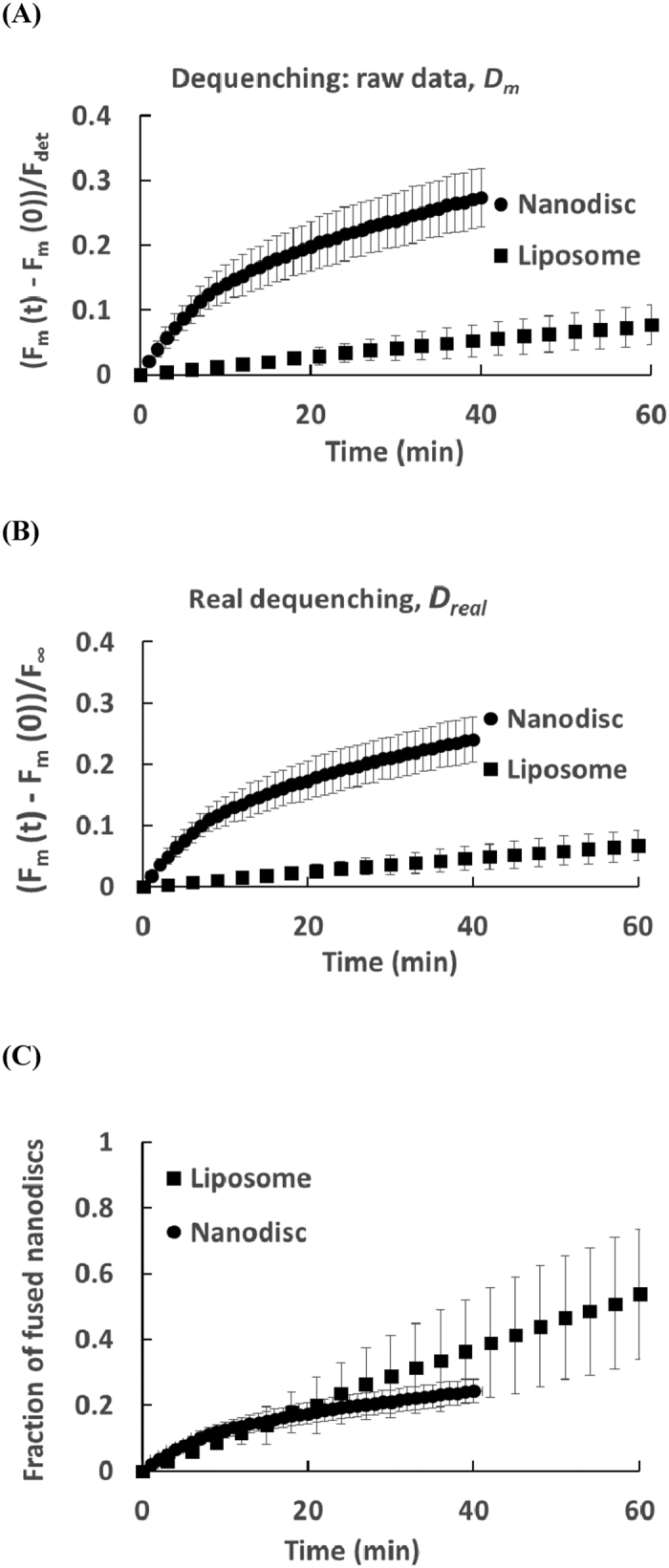
Quantitative comparison of liposome-liposome and liposome-nanodisc SNARE-induced fusion. (**A**) The dequenching after normalization by the fluorescence after addition of DDM, used to represent the kinetics fusion between t-liposomes vs. v-liposomes (squares) and t-liposomes vs. v-nanodiscs (discs), is presented without any correction. The experiments are performed at 37 °C. (**B**) The curves are corrected using *α*_*DDM*_(37 °C) and Eq. (1). The actual dequenching is lower than the apparent one. (**C**) Fraction of v-liposomes or v-nanodiscs that underwent fusion are determined using *δ* and Eq. (5). This procedure allows the quantification of the number of fusion events that take place (see text). In all panels, error bars are standard deviations on 5 different experiments.

To actually compare both experiments, the ratio of the areas of the fluorescent and the non-fluorescent membranes, λ, must be known. In the liposome-liposome experiment, λ is 1 while, in the liposome-nanodisc experiment, λ~60. *R* is directly equal to *D*_*real*_(*t*)/*D*_*all*_. The result is presented in Fig. 5C. It turns out that even though the measured fusion rate seems much faster for the liposome-nanodisc experiment, both curves initially increase linearly and almost overlap during the first 10 minutes, the slope being slightly larger for the liposome-nanodisc case; then, after 20 minutes, the nanodisc-liposome experiment becomes overall less efficient than the liposome-liposome one in terms of fraction of fused membranes.

One interesting feature that can be deduced is the efficiency of collisions for the fusion reaction. Following the standard Smoluchowski approach^13^, the total collision rate for each v-liposome or v-nanodisc with a t-liposome can be approximated by:


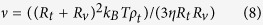


*R*_*t*_ and *R*_*v*_ are the respective hydrodynamic radius of the t-SNARE liposome and the v-SNARE carrier, *ρ*_*t*_ is the concentration of t-liposomes, *η* is the viscosity of the solution (close to that of water) and *k*_*B*_*T* the thermal energy. In our case, the hydrodynamic radius is ~25 nm for liposomes and ~4.5 nm for the nanodiscs which leads to a collision rate between 350 and 400 collisions per second in both the liposome-liposome experiment and the liposome-nanodisc experiment. The initial slope of the corrected dequenching curve (~1/4000 seconds for liposome-liposome and ~1/3000 seconds for liposome-nanodisc) indicates that, in both cases, only one collision per 1–2 million generates fusion. This low fusion efficiency is consistent with that previously determined^14^.

## Discussion

The above results show that the fraction of collisions leading to fusion is extremely low in both liposome-liposome and liposome-nanodisc fusion. However, the shape of the curves are very different, the liposome-liposome experiment has an almost constant rate of fusion, while the rate of liposome-nanodisc fusion decreases with time (Fig. 5C). This is probably because, on average, only 2 v-SNAREs are present on each nanodisc and there are ~50 outward facing v-SNAREs on each v-liposomes. Since ~25 t-SNAREs are outward facing on each t-liposome, after a nanodisc has fused with a t-liposome, all v-SNAREs have assembled with a t-SNARE and the nanodisc cannot undergo a second round of fusion. Hence, the fraction of fusion-potent nanodisc decreases as the fusion processes advances. This explains the decrease of the fusion rate with time. On the contrary, after a v-liposome fuses with a t-liposome, 25 v-SNAREs are still available and the v-liposome is still fusion potent. Thus, all v-liposomes are fusion potent until they undergo at least two rounds of fusion. This explains the constant fusion rate.

A direct consequence of the capability of the nanodisc to undergo one and only one round of fusion is that the nanodisc-liposome curve should plateau at 1 in Fig. 5C. It seems to actually plateau below 1 (around 0.3), suggesting that not all nanodisc are able to fuse with t-liposomes. This may be because some v-SNAREs are not accessible or active on the nanodiscs. In spite of this limited activity, the initial fusion rate of the nanodisc-liposome assay is about 50% larger than that of the liposome-liposome assay. Therefore, for usual lipid mixing fusion assays for which the final *R* does not exceed a few tenth, we strongly recommend to use nanodisc instead of liposomes for two reasons: i. the membrane area ratio, *λ*, is much larger for nanodisc resulting in a larger dequenching and a better fluorescence signal; ii. The initial fusion rate is higher with the nanodisc in spite of the lack of activity of some v-SNAREs. In the rare cases when several rounds of fusion have to be studied, the use of liposomes instead of nanodiscs is required.

To conclude, the standardization presented here provides a direct method to quantitatively compare experiments performed in the lipid mixing assay under conditions that are not necessarily the same. The addition of detergent at the end of the experiment allows to obtain the maximal fluorescence intensity provided that the fluorescence intensity in presence of detergent is corrected by factors that both depend on its nature and on the temperature (*α*_*det*_(*T*)). In order for fusion efficiencies of different systems to be compared, the fluorescence dequenching, which highlights fusion but is not directly comparable for different systems, should moreover be converted to the fraction of membranes that underwent fusion. This can now be done, for different temperatures, provided that the ratios of the surfaces of both types of objects are known. This shows a good quantification requires a correct characterization of the membrane dimensions. Our corrections are only valid for the case of NBD and Rhodamine dyes but a similar calibration could be done for other FRET pairs.

## Methods

### Material

POPC (1-palmitoyl-2-oleoyl-sn-glycero-3-phosphocholine), NBD-DOPE (1,2-dioleoyl-sn-glycero-3-phosphoethanolamine-N-(7-nitro-2-1,3-benzoxadiazol-4-yl) and Rho-DOPE (1,2-dioleoyl-sn-glycero-3-phosphoethanolamine-N-(lissamine rhodamine B sulfonyl) were purchased from Avanti Polar Lipids. β -OG (Octyl β-D-glucopyranoside) and DDM (n-Dodecyl β-D-maltoside) were purchased from Sigma-Aldrich in powder. Stock solutions (DDM 5% wt/wt or β -OG 5% wt/wt) were prepared by dissolving 50 mg of DDM or β -OG in 1 mL of pure water. Triton (Triton™ X-100) was purchased from Sigma Aldrich. A ready to use solution (Triton 4% vol/vol) was prepared by diluting 40 uL of Triton in 1 mL of pure water.

### Protein expression and purification

t-SNARE complexes (rat Syntaxin 1 A and mouse SNAP25, expression vector pTW34), mouse VAMP2 (pet-SUMO-VAMP2) and MSP1E3D1 (pMSP1E3D1, purchased from Addgene Inc.) were expressed and purified as described before^9^,^10^,^15^,^16^,^17^.

### Liposome and nanodisc preparation

Protein free liposomes were formed in a standard manner by extrusion. In brief, POPC, NBD-DOPE and Rho-DOPE were mixed in stock chloroform solutions, in molar ratios varying from 0% to 1.5% of the total lipids for both fluorescent lipids, in a clean glass test tube. The lipids were then dried by a flow of nitrogen in the bottom of the tube. After desiccating the tubes under vacuum for at least two hours to enable the evaporation of residual chloroform, the lipids were rehydrated with an HEPES buffer (25 mM HEPES, 100 mM KCl, pH = 7.4) under agitation for ten minutes, in order to have a given lipid concentration (18 mM or 1 mM according to the experiment). The suspension was then vigorously vortexed, frozen in liquid nitrogen and thawed five times, and extruded 21 times through a polycarbonate membrane containing 50 nm sized pores (Avanti Polar Lipids) using a mini extruder (Avanti Polar Lipids). Proteoliposomes and nanodiscs were prepared as described previously^9^,^10^,^15^,^18^.

### Fluorescence measurements, dilution experiments and lipid mixing assay

For all the fluorescent measurements, a 96-well plate reader (SpectraMax M5e, Molecular Devices) was used. The NBD was excited at 460 nm and the emission was read at 538 nm. The 96-well plates were clear (#353072, Falcon) in order for the fluorescence to be read from the bottom, to prevent a reading artifact due to possible condensation of water on the lid at the highest temperatures. For the lipid mixing assay, non-fluorescent proteo-liposomes containing t-SNAREs, t-liposomes, are mixed with either fluorescent proteo-liposomes or nanodiscs containing v-SNARE (v-liposomes and v-nanodiscs respectively). The protein to lipid ratios and t-liposomes to v-liposome (or v-nanodiscs) ratios are specified in the text for each situation.

## Additional Information

**How to cite this article:** François-Martin, C. and Pincet, F. Actual fusion efficiency in the lipid mixing assay - Comparison between nanodiscs and liposomes. *Sci. Rep.* **7**, 43860; doi: 10.1038/srep43860 (2017).

**Publisher's note:** Springer Nature remains neutral with regard to jurisdictional claims in published maps and institutional affiliations.

## Figures and Tables

**Table 1:** Ratios α_det_(T) of the real fluorescence at infinite dilution to that with detergent (see Eq. 1) at temperatures ranging from 27 °C to 47 °C.

*T*		27 °C	32 °C	37 °C	42 °C	47 °C
*α*_*det*_(*T*)	DDM	1.19 ± 0.02	1.19 ± 0.02	1.22 ± 0.03	1.23 ± 0.03	1.24 ± 0.01
	Triton	1.71 ± 0.03	1.73 ± 0.03	1.79 ± 0.03	1.80 ± 0.01	2.01 ± 0.02
	β-OG	1.64 ± 0.03	1.65 ± 0.03	1.71 ± 0.03	1.73 ± 0.04	1.74 ± 0.03
